# Restless legs syndrome in end stage renal disease patients undergoing hemodialysis

**DOI:** 10.1186/s12883-019-1265-y

**Published:** 2019-03-29

**Authors:** Xiao-Wei Lin, Jun-Fang Zhang, Meng-Yao Qiu, Ling-Yan Ni, Hong-Lei Yu, Sheng-Han Kuo, William G. Ondo, Qing Yu, Yun-Cheng Wu

**Affiliations:** 1 Department of Neurology, Shanghai General Hospital, Shanghai Jiao Tong University School of Medicine, Shanghai, 200080 People’s Republic of China; 2 Department of Nephrology, Shanghai General Hospital, Shanghai Jiao Tong University School of Medicine, Shanghai, 200080 People’s Republic of China; 3 Department of Neurology, College of Physicians and Surgeons, Columbia University, New York, NY USA; 4 Department of Neurology, Methodist Neurological Institute, Weill Cornell Medical School, Houston, TX USA

**Keywords:** RLS, ESRD, Female, Alcohol intake, Sleep disorder

## Abstract

**Background:**

The prevalence of Restless legs syndrome (RLS) in End Stage Renal Disease (ESRD) patients is higher than that in the general population. However, the associations of RLS within the ESRD population are inconsistent and RLS is usually neglected in dialysis centers, although it impairs the life quality among ESRD patients. We aim to investigate the prevalence of RLS in patients with ESRD undergoing maintenance hemodialysis and evaluate the risk factors of developing RLS and the effect of RLS on quality of life among ESRD patients.

**Methods:**

ESRD patients undergoing maintenance hemodialysis in Shanghai General Hospital dialysis unit from July 2016 to October 2016 were enrolled in the study. RLS was diagnosed according to the criteria of the International Restless Legs Syndrome Study Group (IRLSSG). IRLSSG Severity Scale was used to evaluate the severity of RLS. Pittsburgh Sleep Quality Index (PSQI) was used to evaluate sleep quality, and Hospital Anxiety and Depression Scale (HADS) was used to estimate anxiety and depression. Serologic and historic variables were analyzed to determine predictors of RLS in the ESRD population.

**Results:**

A total of 137 ESRD patients were enrolled. The prevalence of RLS among the ESRD patients was 20.44%. The risk of RLS was increased significantly in females (OR = 2.729, *p* = 0.032) and daily alcohol drinkers (OR = 4.716, *p* = 0.022). RLS increased the risks of sleep disorders (25/28, 89.3% vs 73/109, 67.0%, *p* = 0.02) and sedative hypnotics intake (7/28, 25.0% vs 10/109, 9.2%, *p* = 0.047) and impaired the sleep quality (7/109 vs 11/28, *p* = 0.001) according to PSQI sum scores.

**Conclusion:**

A high RLS prevalence among the ESRD patients undergoing hemodialysis was confirmed. ESRD patients who are women and drinking alcohol have a higher risk of RLS. The sleep quality was significantly impaired and sleeping medication use was more common among the ESRD patients with RLS.

## Background

Restless legs syndrome (RLS) is a common neurological disorder, which can be primary or associated with other conditions. RLS symptoms are characterized by the uncomfortable or abnormal sensations inside the legs or arms, associated with an urge to move the limbs. The symptoms usually occur at rest and at night, and can be temporarily relieved by movement [1]. RLS has an adverse impact on the quality of life and can be associated with mood disorders such as anxiety and depression [2].

In the general population, the prevalence of RLS ranges from 3 to 9%, depending on the age and gender [3]. However, the prevalence of RLS in End Stage Renal Disease (ESRD) characterized as permanent loss of renal function requiring renal replacement therapy or dialysis, is 6.6–70%, which is much higher than the general population [4].

RLS in ESRD patients has been variably reported to be associated with female gender [5, 6], duration of dialysis [4, 7], diabetes mellitus [4, 8–10], iron deficiency anemia [4, 10, 11], parathyroid hormone [12], increased body mass index (BMI) [13], and increased homocysteine [14]. However, results are very inconsistent. [10, 15].

The occurrence of RLS among the ESRD patients impairs quality of life compared with the ESRD patients without RLS, possibly due to the poor sleep quality, insomnia or depression [9, 16]. Therefore, further studies are needed to determine the prevalence, risk factors and impact of RLS in ESRD patients.

The aim of the study was to investigate the prevalence of RLS in patients with ESRD undergoing maintenance hemodialysis in Shanghai General Hospital, China. We also aimed to evaluate the risk factors of developing RLS and to investigate the effect of RLS on quality of life among ESRD patients.

## Methods

### Study subjects

All ESRD patients undergoing long-term hemodialysis in the Shanghai General Hospital dialysis unit from July 2016 to October 2016 were approached for our study. Patients will be excluded when they were less than 3 months with hemodialysis duration, less than 18 years old, pregnant, Parkinson’s disease, type 1 diabetes mellitus and peripheral neuropathy. The protocol was approved by the ethics committee of Shanghai General Hospital, and written informed consents were obtained.

### Study design

Of the 161 ESRD patients enrolled in our study, 24 patients were excluded, including two patients with the less than 3 months of hemodialysis duration, twenty-one patients declined to participate, and one patient had type 1 diabetes mellitus, leaving 137 patients.

All the patients are diagnosed with the detailed history and International Restless Legs Syndrome Study Group (IRLSSG) scale which were carried out by neurologists through face-to-face interviews. The demographic questionnaire included gender, age, height, weight, cause of ESRD, hemodialysis data, other diseases, medication history, family history, personal history, etc. Furthermore, every participant was requested to complete the Pittsburgh Sleep Quality Index (PSQI) and Hospital Anxiety and Depression Scale (HADS).

The PSQI is used to evaluate the sleep quality of ESRD patients in seven parts: subjective quality of sleep, sleep latency, sleep duration, habitual sleep efficiency, presence of sleep disturbances, use of hypnotic-sedative medication and daytime dysfunction [17]. Participants with PSQI total scores of ≥5 were considered to have sleep disorders in our study.

The HADS consists of two subscales (HADS-A and HADS-D), which are composed of seven anxiety items and seven depression items respectively. The four options of every item were scored 0–3. The total subscale scores of 8–10 indicate doubtful anxiety or depression and sores of 11 or more indicate definite anxiety or depression [18].

The presence of RLS was assessed using the minimum diagnostic criteria recommended by the International Restless Legs Syndrome Study Group (IRLSSG), which consists of four questions: (1) an urge to move the legs, usually accompanied or caused by uncomfortable and unpleasant sensations in the legs; (2) the urge to move or unpleasant sensations begin or worsen during periods of rest or inactivity, such as lying or sitting; (3) the urge to move or unpleasant sensations are partially or totally relieved by movement, such as walking or stretching, at least as long as the activity continues, and (4) the urge to move or unpleasant sensations are worse in the evening or night than during the day or only occur in the evening or night [1]. Participants with four “yes” replied to the four aforementioned questions were diagnosed with RLS. RLS participants completed the IRLSSG Severity Scale with 10 questions for evaluating severity of the RLS symptoms [19]. Patients were classified into four groups with mild (0–10 scores), moderate (11–20 scores), severe (21–30 scores) and very severe (31–40 scores) RLS.

Laboratory blood tests included blood urea nitrogen (BUN), pre-dialysis creatinine, post-dialysis creatinine, albumin, alkaline phosphatase (AKP), calcium, phosphate, hemoglobin, serum iron, ferritin, transferrin, total iron binding capacity (TIBC) and parathyroid hormone (PTH), which were tested within a week of the interview.

### Statistical analysis

The data were analyzed via SPSS version 17.0 (SPSS Inc., Chicago, IL, USA). T-test or Mann-Whitney U test was used for continuous variables and Chi-square test was used for categorical variables. The continuous data are presented as mean and standard deviation (normal distribution) or median and quartiles (non-normal distribution). Multivariate analysis was performed by binary logistic regression analysis, which allows adjustment for confounding factors, and all variables that had *p* < 0.1 in univariate analysis were used as independent variables. *p* < 0.05 was considered statistically significant.

## Results

The data of 137 patients were analyzed in our study. There were 74 males (54%) and 63 females (46%). The mean age was 55.47 ± 12.76 years old. The mean duration of hemodialysis was 98.35 ± 71.66 months. The causes of ESRD included chronic glomerulonephritis (*n* = 89, 65.0%), hypertensive nephrosclerosis (*n* = 17, 12.4%), diabetic nephropathy (*n* = 6, 4.4%), polycystic kidney disease (*n* = 8, 5.8%), drug induced nephropathy (*n* = 5, 3.6%), vascular kidney disease (*n* = 6, 4.4%), autoimmune nephritis (*n* = 2, 1.5%), and unknown etiology (*n* = 4, 2.9%). RLS was found in 28 patients (20.44%). Only one of them had been previously diagnosed with RLS and received *pramipexole* treatment. There were 6 patients with mild RLS, 15 patients with moderate RLS, 7 patients with severe RLS and no patients with very severe RLS, based on the IRLSSG rating scale.

Demographic data and routine laboratory blood test data are summarized in Table 1 and Table 2. According to the univariate analyses, BUN (*p* = 0.014) and serum iron (*p* = 0.034) were significantly higher in RLS positive group. Female sex (*p* = 0.08), post-dialysis systolic pressure (*p* = 0.053) and alcohol intake (*p* = 0.069) seems to be the risk factors of RLS, but without statistically significance. Age, body mass index (BMI), weeks on hemodialysis, frequency of hemodialysis, dialysis solution temperature, diabetes mellitus Type 2, family history of RLS, smoking, tea intake, coffee intake and other routine laboratory blood test data including creatinine, albumin, alkaline phosphatase (AKP), calcium, phosphate, hemoglobin, ferritin, transferrin, total iron binding capacity (TIBC), parathyroid hormone (PTH), URR, and Kt/v, showed no relationship with RLS (*P* Value > 0.05).

**Table 1:** The demographic data of the ESRD patients with and without RLS

		Patients with ESRD, N (%)		
Variables		RLS –(*n* = 109)	RLS +(*n* = 28)	*p* value
Female		46 (42.2%)	17 (58.8%)	0.08
Age (year)		55.83 ± 13.20	54.04 ± 10.40	0.505
Educational Attainment	< Middle school	7 (6.4%)	4 (23.5%)	
	Middle school or vocational	70 (64.2%)	16 (57.1%)	
	High school or higher	32 (29.4%)	8 (28.6%)	0.548
BMI (kg/m2)	Underweight (< 18.5)	18 (16.5%)	6 (21.4%)	
	Normal Range (18.5-23.9)	67 (61.5%)	18 (64.3%)	
	Overweight (24.0-27.9)	20 (18.3%)	3 (10.7%)	
	Obese (≥28.0)	4 (3.7%)	1 (3.6%)	0.346
Duration of Hemodialysis (week)		90 (25-132)	113.5 (39-159.75)	0.224
Frequency of Hemodialysis	3 times/week	102 (93.6%)	25 (89.3%)	0.438
Dialysis Solution Temperature	36 °C	52 (47.7%)	14 (50.0%)	
	36.5 °C	46 (42.2%)	10 (35.7%)	
	37 °C	11 (10.1%)	4 (15.3%)	0.991
Pre-dialysis Systolic Pressure (mmHg)		131.51 ± 20.44	125.36 ± 21.21	0.161
Pre-dialysis Diastolic Pressure (mmHg)		78.97 ± 11.46	77.86 ± 12.80	0.655
Post-dialysis Systolic Pressure (mmHg)		122.89 ± 19.88	115 ± 15.46	0.053
Post-dialysis Diastolic Pressure (mmHg)		74.60 ± 11.71	72.32 ± 11.43	0.358
Diabetes Mellitus		28 (25.7%)	9 (32.1%)	0.734
Hypertension		89 (81.7%)	24 (86.0%)	0.783
Family history	non	94 (86.2%)	22 (78.6%)	
	Hypertension	10 (9.2%)	3 (10.7%)	
	Polycystic kidney	2 (1.8%)	3 (10.7%)	
	Diabetes mellitus	3 (2.8%)	0 (0%)	0.151
Smoking	Daily and regularly	28 (25.7%)	9 (32.1%)	0.493
Alcohol intake	Daily and regularly	7 (6.4%)	5 (17.9%)	0.069
Tea intake	Daily and regularly	43 (39.4%)	9 (32.1%)	0.477
Coffee intake	Daily and regularly	18 (16.5%)	6 (21.4%)	0.58

*Abbreviations*: *ESRD* end stage renal disease, *RLS* restless legs syndrome, *RLS-* without RLS, *RLS+* with RLS, *BMI* body mass index

**Table 2:** Laboratory blood test of the ESRD patients with and without RLS

		Patients with ESRD, N (%)		
Variables		RLS – (n = 109)	RLS + (n = 28)	*p* value
Blood Urea Nitrogen	mmol/L	27.48 (23.31-31.35)	29.33 (27.3-34.2)	0.014
Pre-dialysis Creatinine	mg/dl	1037 (874.5-1204.5)	1053 (900-1193.5)	0.852
Pro-dialysis Creatinine	mg/dl	350 (276-418)	327 (247-462)	0.378
D-value of Creatinine	mg/dl	685 (559.5-791.5)	699 (641.25-805.75)	0.370
Calcium	mg/dL	2.31 ± 0.26	2.32 ± 0.26	0.744
Phosphorus	mg/dL	1.90 ± 0.47	2.11 ± 0.70	0.155
Ca*P product	mg2/dL^2^	4.41 ± 1.30	4.92 ± 1.75	0.157
Albumin	g/dL	39.24 ± 4.24	39.70 ± 6.29	0.650
Alkaline Phosphatase	U/L	73 (54-93.5)	75 (61-98.75)	0.523
Hemoglobin	g/L	100.13 ± 15.54	105.43 ± 22.1	0.240
Serum Iron	μmol/L	13.2 (9.45-16.3)	15.41 (12.5-18.0)	0.034
Ferritin	μg/L	194 (68.85-335.3)	122.5 (53.8-232.99)	0.371
Transferrin	g/L	2.11 (1.83-2.36)	2.14 (1.78-2.45)	0.953
TIBC	μmol/L	47.02 ± 8.49	48.51 ± 9.67	0.424
Parathyroid Hormone	pg/mL	203.9 (65.91-472.1)	258.3 (62.95-574.1)	0.522
Kt/V		1.53 ± 0.61	1.49 ± 0.34	0.783
URR	%	70.19 ± 8.15	70.27 ± 8.05	0.967

*Abbreviations*: *ESRD* end stage renal disease, *RLS* restless legs syndrome, *RLS-* without RLS, *RLS+* with RLS, Ca*P product calcium phosphorus product, *TIBC* total iron-binding capacity, Kt/V is a number used to quantify hemodialysis (K, dialyzer clearance of urea; t, dialysis time; V, volume of distribution of urea, approximately equal to patient’s total body water); URR, urea reduction ratio

All variables that had *p* < 0.1 in univariate analysis, including BUN (*p* = 0.014), serum iron (*p* = 0.034), female (*p* = 0.08), post-dialysis systolic pressure (*p* = 0.053) and alcohol intake (*p* = 0.069), were included in multivariable regression analysis to identify independent risk factors of RLS among ESRD patients. We found that only female sex (OR 2.729, 95% CI 1.087–6.849, *p* = 0.032) and daily alcohol intake (OR 4.716, 95% CI 1.247–17.837, *p* = 0.022) were significantly independent risk factors.

We also compared sleep quality, depression, anxiety and medications between ESRD patients with RLS and those without RLS (Table 3). RLS patients had significantly higher proportion of sleep disorders (89.3% vs 67.0%, *p* = 0.02) and lower sleep quality (11 vs 7, *p* = 0.001) according to PSQI total scores. The proportion of sedative hypnotics intake (25.0% vs 9.2%, *p* = 0.047) was significantly increased in the patients with RLS.

**Table 3:** Sleep quality, depression, anxiety and medications between RLS- and RLS+

		Patients with ESRD, N (%)		
		RLS- (n = 109)	RLS+ (n = 28)	*P*- value
PSQI		7 (4-10)	11 (5.25-13)	0.001
Sleep disorder		73 (67.0%)	25 (89.3%)	0.020
HADS-D		5 (1-9)	3 (1-7)	0.622
Depression	No symptom	70 (64.2%)	22 (78.6%)	0.251
	Suspicious	20 (18.3%)	1 (3.6%)	
	Confirmed	19 (17.4%)	5 (17.9%)	
HADS-A		2 (0-4)	3 (1-6.5)	0.151
Anxiety	No symptom	99 (91%)	25 (89.3%)	0.773
	Suspicious	6 (5.5%)	1 (3.6%)	
	Confirmed	4 (3.7%)	2 (7.1%)	
Medications	Anti-anemia Medicine	85 (78.0%)	23 (82.1%)	0.631
	Sedative hypnotics	10 (9.2%)	7 (25.0%)	0.047

*Abbreviations*: *ESRD* end stage renal disease, *RLS* restless legs syndrome, *RLS-* without RLS, *RLS+* with RLS, *PSQI* Pittsburgh Sleep Quality Index, *HADS-D* Hospital Anxiety and Depression Scale of Depression, *HADS-A* HADS-Anxiety

## Discussion

The prevalence of RLS among the ESRD patients varies widely in different studies. It was 15.8% in Iran, 22% in Japan, 25.3% in Taiwan, 26.6% in Greek, and 52.6% in Brazil [4, 5, 7, 20, 21]. However, it has not been studied about the prevalence in mainland China. The discrepancy of RLS prevalence among ESRD patients might be influenced by racial heterogeneity, demographic difference [10], diagnostic criteria of RLS [22], differences in dialysis techniques, and methods of study. In our study, all the patients were long-term residence in Shanghai of China. RLS diagnosis was made according to the 4 minimum diagnostic criteria of IRLSSG. Our prevalence of RLS among ESRD patients (20.44%) was similar to some large scale studies [4, 5, 10] and significantly higher than among the general Chinese population (7.2%) [23].

Our result reveals that RLS is more common in female ESRD patients (OR = 2.729, *p* = 0.032), similar to some other studies [5, 8, 24]. In general population studies, RLS is consistently more common in females [23, 25], although one German study found this was explained by previous pregnancies, such that null-parous women had similar RLS rates as men [25, 26]. The high level of estrogen during pregnancy could trigger RLS [27]. Interestingly, the concentration of estrogen level is usually high in advanced kidney failure, potentially explaining this [28].

In our study, the ratio of alcohol intake among RLS patients was higher than patients without RLS, which was not significant (*p* = 0.069) in univariate analysis but was significant (*p* = 0.022) in the multivariable backward logistic regression model. Regular alcohol intake increased the risk of developing RLS by 4.72 times in our ESRD patients. One study demonstrated that alcohol intake raised the risk of developing RLS in the general population [29]. However, a potentially protective effect of alcohol on the risk of RLS was found in several studies [30, 31]. These contradictory conclusions can be argued that moderate drinking could reduce the risk of RLS while excessive alcohol consumption could produce and aggravate RLS symptoms [32]. Therefore, additional studies are needed to clarify the role of alcohol in ESRD patients with RLS.

In contrast to some studies [4, 8–10], we found no significant association between diabetes mellitus type 2 and RLS among ESRD patients. It is not clear whether diabetes mellitus itself or diabetic peripheral neuropathy is the dominant risk factor [33]. Some other studies found that there are no correlationship between diabetes mellitus and the occurrence of RLS in ESRD patients [34, 35]. While a multicenter study in Taiwan showed that having type 2 diabetes is associated with RLS [4].

No significant association was found between iron deficiency and anemia, or iron deficiency and RLS among ESRD patients in our study. Iron-Deficiency Anemia (IDA) and the relevant blood parameters were reported as significant risk factors of RLS among the patients with ESRD [4, 10, 11]. Furthermore, the symptoms of RLS have improved with high-dose iron dextran therapy among the ESRD patients, which also suggested that IDA could induce RLS [36] . Other studies showed no relationship between IDA and RLS in ESRD patients [5, 8, 12].

In our study, we didn’t find any difference between two groups regarding to Parathyroid hormone (PTH). Previous studies show mixed results. One previous study found PTH levels to be lower in the ESRD patients with RLS than those without RLS [12]. On the contrary, recent studies indicated that PTH was an independent risk factor [5, 37], which is supported by the finding that parathyroidectomy improved RLS in ESRD patients in one study [20]. Nevertheless, some studies suggested that there is no association between PTH and RLS in the ESRD patients [4, 10, 14]. Therefore, further large studies are needed to clarify the relationship between PTH and RLS in ESRD patients.

In our study, we found no statistical difference of BMI between ESRD patients with RLS and those without RLS (*p* = 0.346), which was similar to some other studies of RLS among patient with ESRD. In contrast to the results of our study, some studies indicated that the increasing BMI led to significantly higher odds of developing RLS both in patients with ESRD and in general population [30, 38].

Our study found no significant association between RLS and the duration of hemodialysis, blood pressure on hemodialysis, smoking, tea or coffee intake and blood markers in multivariable backward logistic regression analysis, similar to some previous studies [10, 15, 34, 38].

Previous studies indicated that ESRD patients with RLS suffered from poorer sleep quality than those without RLS [4, 14, 39]. Our study also found significant associations between RLS and sleep-related problems, including prolonged sleep latency, night awakening, shorten sleep duration and sleeping medication use. And also, RLS had an association with impairment of sleep quality among the ESRD patients. Sleep disorders occurred more frequently and sleep quality was poorer among ESRD patients with RLS than those without RLS. Moreover, ESRD patients with RLS were more likely to require sedative hypnotics to improve their sleep quality.

Depression prevalence in hemodialysis patients was reported to be 15–30%, depending on the method of depression assessment and demographics [40]. The incidence of anxiety in hemodialysis patients ranged from 12 to 52% [41]. In our study, the incidence of confirmed depression in ESRD patients with RLS of 17.4% was similar to the previous studies. However, there was no significant difference in depression between ESRD patients with RLS and without RLS. The incidence of confirmed anxiety in ESRD patients with RLS (7.1%) in our study was lower than those in other studies, and no significant difference of anxiety existed between ESRD patients with RLS and without RLS. Due to the face-to-face interviews in the public area, patients were likely to have misgivings about giving the real answers to the HADS questionnaire. This may be responsible for the irrelevancy between RLS and depression/anxiety.

Several limitations of this study should be noted. Firstly, the cross-sectional design and descriptive nature do not allow for conclusions about the direction of theses relationships. Secondly, this is a single-center study with comparatively small number of participants, which decreases the statistical power. Thirdly, we didn’t make formal evaluations to look for peripheral neuropathy. Lastly, in our present study, we only included hemodialysis patients and did not include peritoneal dialysis patients, which partially limits the comparison with previous studies.

## Conclusion

Our study confirmed a high incidence of RLS among the ESRD patients undergoing maintenance hemodialysis. Among ESRD patients, the risk of RLS was significantly increased in females and alcohol users. The sleep quality was significantly impaired and sleeping medication use was more frequent among the ESRD patients with RLS. High prevalence of RLS among ESRD patients suggests the need for clinicians to improve awareness and recognition of RLS among ESRD patients. Although the risk factors of RLS among ESRD patients were identified by many studies, the results are still disputable. Therefore, more multicenter studies are needed to confirm the various risk or protective factors in the future.

## Abbreviations

AKP: Alkaline phosphatase
BMI: Body mass index
BUN: Blood urea nitrogen
ESRD: End stage renal disease
HADS: Hospital anxiety and depression scale
IDA: Iron-Deficiency anemia
IRLSSG: International restless legs syndrome study group
PSQI: Pittsburgh sleep quality index
PTH: Parathyroid hormone
RLS: Restless legs syndrome
TIBC: total iron binding capacity
URR: urea reduction ratio
